# A dataset of multi-contrast unbiased average MRI templates of a Parkinson's disease population

**DOI:** 10.1016/j.dib.2023.109141

**Published:** 2023-04-12

**Authors:** Victoria Madge, Vladimir S Fonov, Yiming Xiao, Lucy Zou, Courtney Jackson, Ronald B Postuma, Alain Dagher, Edward A Fon, D Louis Collins

**Affiliations:** a Montreal Neurological Institute, McGill University, Montreal, QC, Canada; b Concordia University, Montreal, QC, Canada

**Keywords:** Magnetic resonance imaging (MRI), Susceptibility-weighted imaging (SWI), Neuromelanin-sensitive imaging, Parkinson's disease, Template, Midbrain, Substantia nigra

## Abstract

Parkinson's disease (PD) is a complex neurodegenerative disorder affecting regions such as the substantia nigra (SN), red nucleus (RN) and locus coeruleus (LC). Processing MRI data from patients with PD requires anatomical structural references for spatial normalization and structural segmentation. Extending our previous work, we present multi-contrast unbiased MRI templates using nine 3T MRI modalities: T1w, T2*w, T1-T2* fusion, R2*, T2w, PDw, fluid-attenuated inversion recovery (FLAIR), susceptibility-weighted imaging, and neuromelanin-sensitive MRI (NM). One mm isotropic voxel size templates were created, along with 0.5 mm isotropic whole brain templates and 0.3 mm isotropic templates of the midbrain. All templates were created from 126 PD patients (44 female; ages=40–87), and 17 healthy controls (13 female; ages=39–84), except the NM template, which was created from 85 PD patients and 13 controls, respectively. The dataset is available on the NIST MNI Repository via the following link: http://nist.mni.mcgill.ca/multi-contrast-pd126-and-ctrl17-templates/. The data is also available on NITRC at the following link: https://www.nitrc.org/projects/pd126/.

**Specifications Table**

**Table:** 

Subject	Health and medical sciences
Specific subject area	Brain MRI templates of Parkinson's disease population
Type of data	Images, figures, volume data
How the data were acquired	3T Magnetic resonance imaging (Siemens 3T Prisma MRI scanner)
Data format	Processed
Description of data collection	Subjects were recruited through the Quebec Parkinson's Network (QPN), PD patients with a PD diagnosis, inclusion and exclusion criteria based on [1], and age- and sex- matched healthy controls. 3T MRI sequences used to make templates include: T1w MPRAGE, multi-echo GRE [2], T2w PD, 2D FLAIR, improved susceptibility weighted imaging (CLEAR-SWI) [3], T1-weighted Turbo Spin Echo (NM) [4]. Multislice MRI was used to create the Neuromelanin template. Raw images went through denoising [5], non-uniformity correction [6], and intensity normalization.
Data source location	Institution: Montreal Neurological Institute (MNI) City/Town/Region: Montreal Country: Canada
Data accessibility	The dataset is available on the NIST MNI Repository via the following link: http://nist.mni.mcgill.ca/multi-contrast-pd126-and-ctrl17-templates/. The data is also available on NITRC at the following link: https://www.nitrc.org/projects/pd126/.

## Value of the Data

• These publicly available templates were created using 126 PD patients while current templates use much smaller cohorts.
• Templates represent averaged anatomical and MRI intensity features of Parkinson's disease, and healthy controls, in nine different MRI contrasts, respectively.
• Data is useful in processing and structural analysis of images from subjects with Parkinson's disease, as there is less anatomical bias when using the PD126 template compared to templates created from cohorts of healthy adults. The PD126 template can be used as a stereotaxic registration target, and both PD126 and CTRL17 templates can be used in deformation-based morphometry analyses.
• Accompanying study-specific templates of 17 healthy controls is advantageous to use over generic templates of healthy adults in group-wise analyses, given that all subjects underwent the same imaging protocol, all images underwent the same processing, and there is no significant difference in age between subjects used for both PD126 and CTRL17 templates [7].

## 1 Data Description

**Fig. 1:**
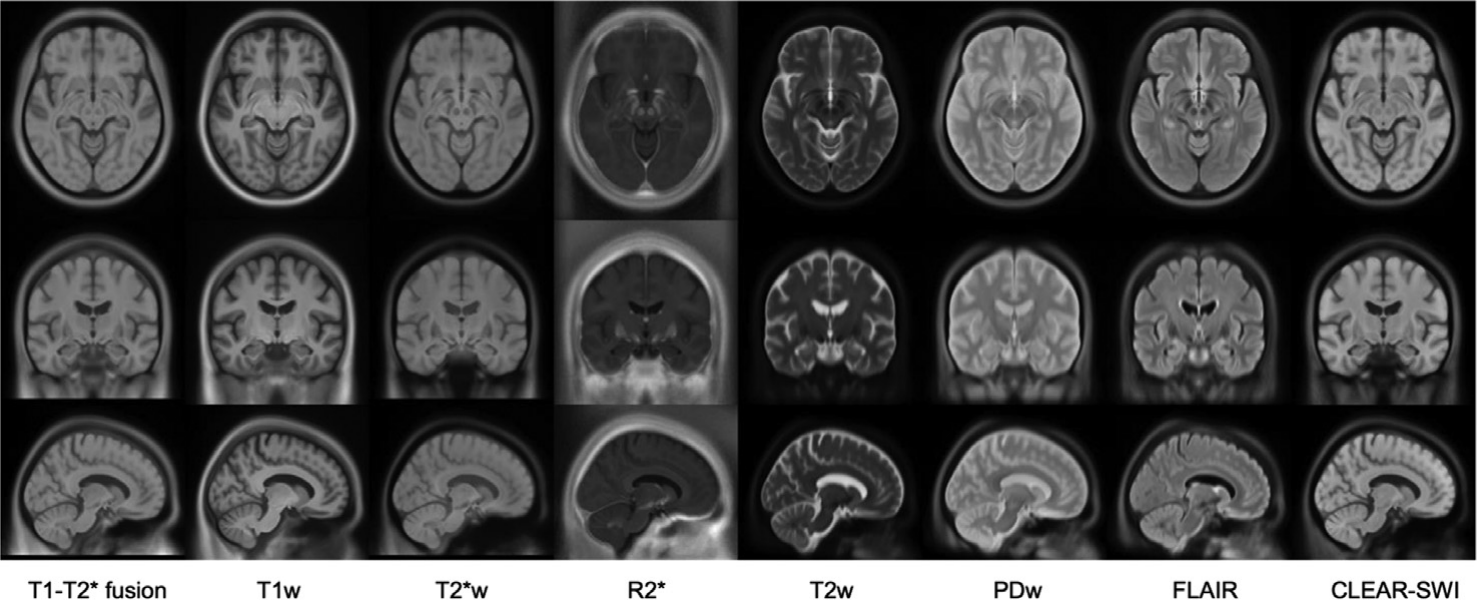
Multi-contrast population of 126 PD patients (voxel size 1 × 1 × 1 mm^3^), showing eight contrasts in columns from left to right: T1-T2* fusion, T1w, T2*w, R2*, T2w, PDw, FLAIR, CLEAR-SWI. The templates are shown in three slices: axial (row 1), coronal (row 2), and sagittal (row 3).

**Fig. 2:**
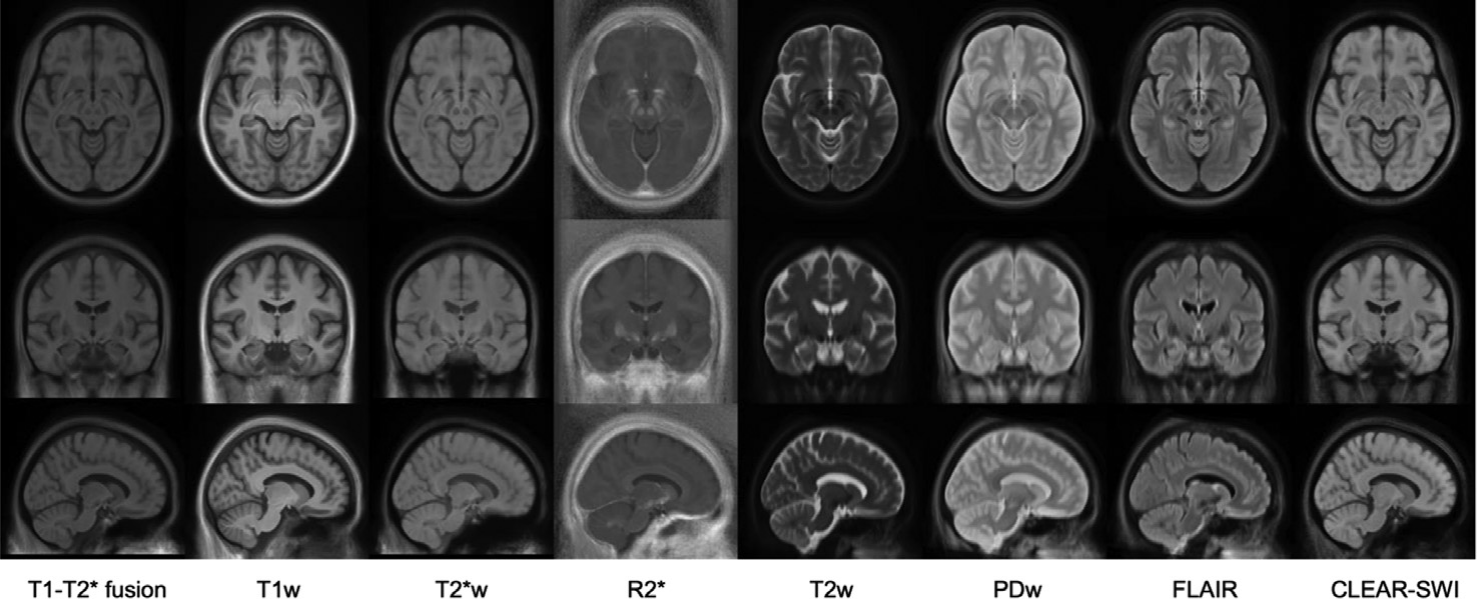
Multi-contrast population of 17 healthy controls (voxel size 1 × 1 × 1 mm^3^), showing eight contrasts in columns from left to right: T1-T2* fusion, T1w, T2*w, R2*, T2w, PDw, FLAIR, CLEAR-SWI. The templates are shown in three slices: axial (row 1), coronal (row 2), and sagittal (row 3).

**Fig. 3:**
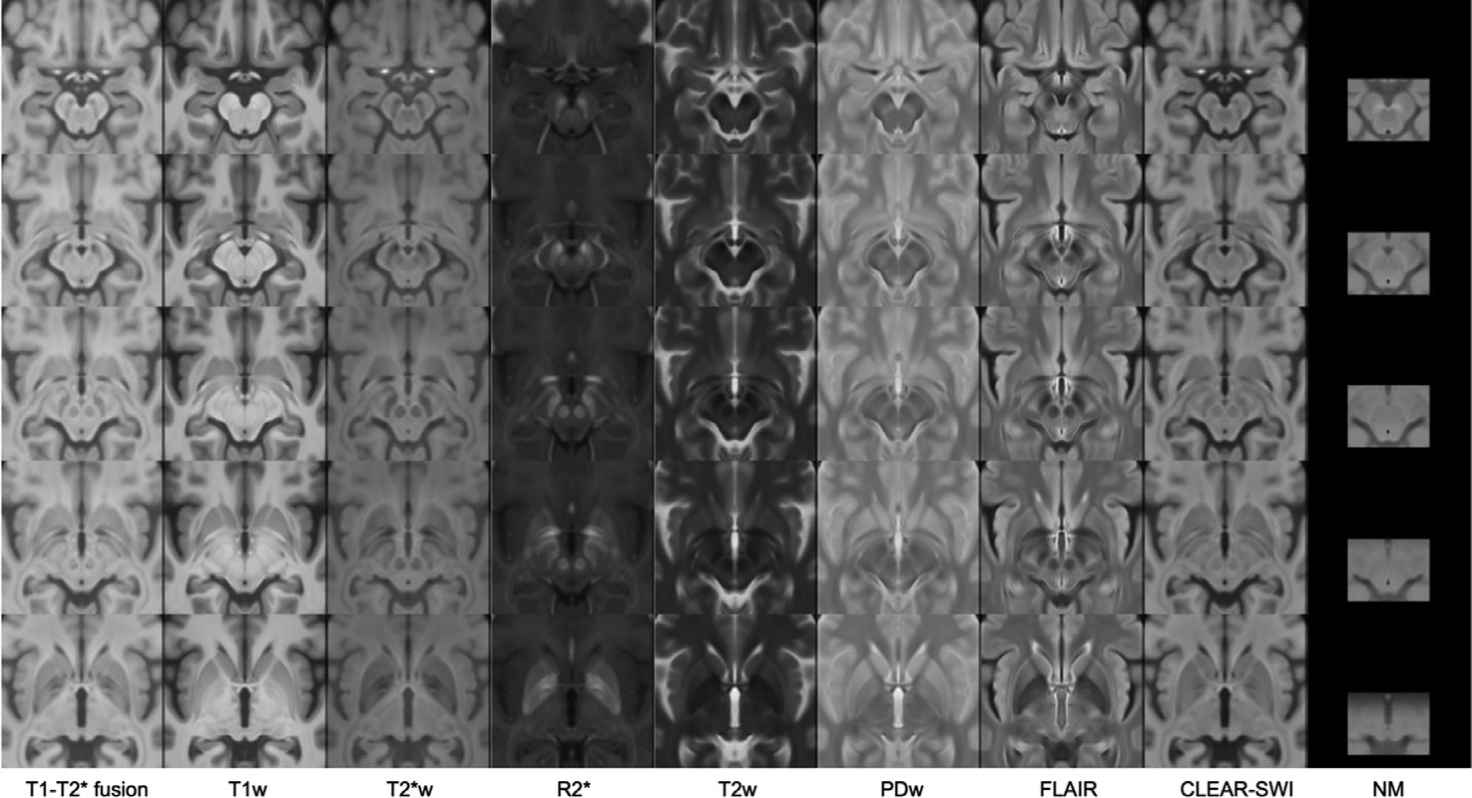
Multi-contrast population of 126 PD patients (voxel size 0.3 × 0.3 × 0.3 mm^3^), showing nine contrasts in columns from left to right: T1-T2* fusion, T1w, T2*w, R2*, T2w, PDw, FLAIR, CLEAR-SWI, NM. The templates are shown as five axial slices from superior to inferior direction (rows from top to bottom at *z* = 87,101,108,115,129 mm in stereotaxic space). Note that the NM template is derived from 85 patients only, and from an NM-sensitive sequence which is not 3D and does not cover the full extent of the brain.

**Fig. 4:**
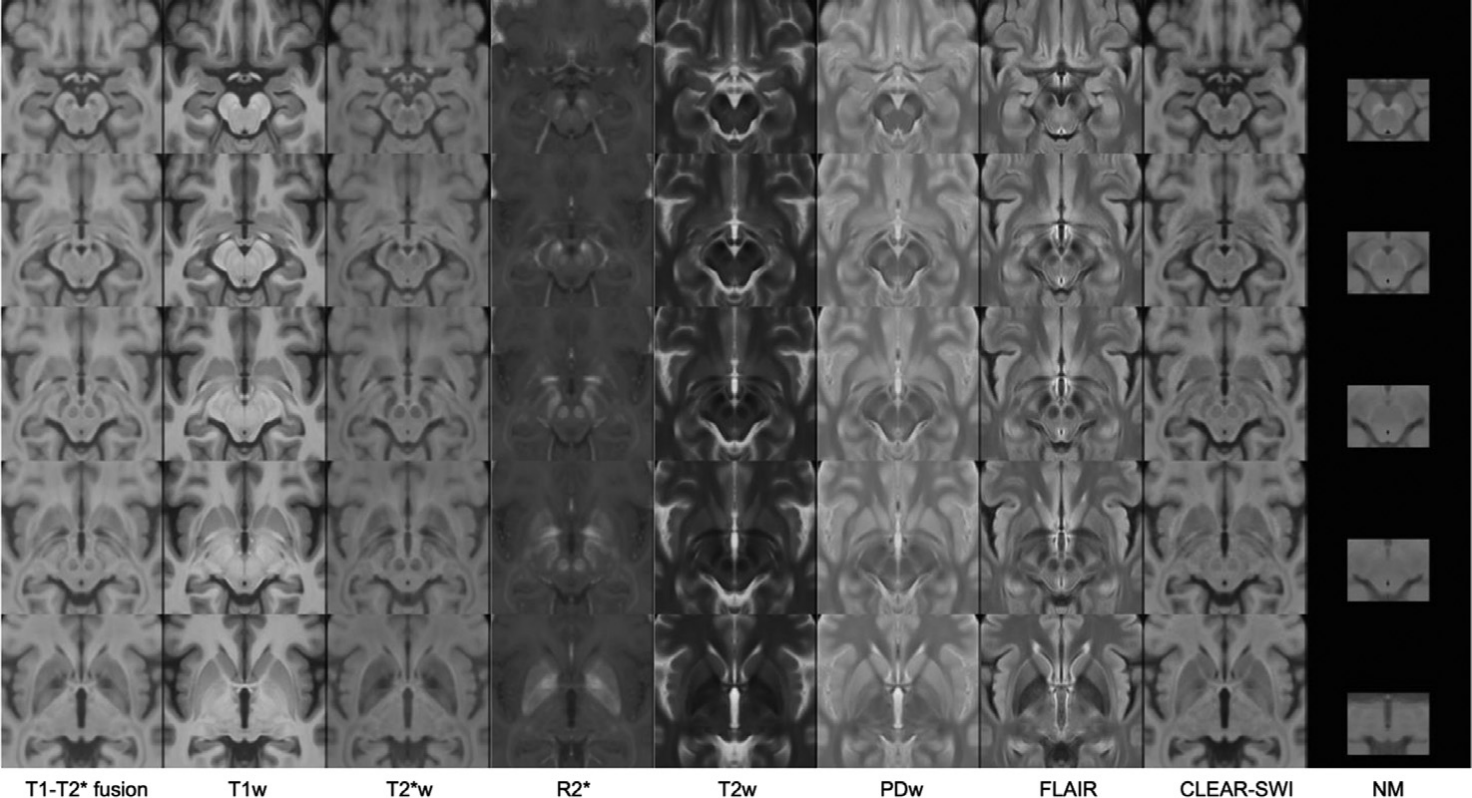
Multi-contrast population of 17 healthy controls (voxel size 0.3 × 0.3 × 0.3 mm^3^), showing nine contrasts in columns from left to right: T1-T2* fusion, T1w, T2*w, R2*, T2w, PDw, FLAIR, CLEAR-SWI, NM. The templates are shown as five axial slices from superior to inferior direction (rows from top to bottom at *z* = 87,101,108,115,129 mm in stereotaxic space). Note that the NM template is derived from 13 subjects only, and from an NM-sensitive sequence which is not 3D and does not cover the full extent of the brain.

Extending our previous work [2], [8], the dataset is a collection of nine multi-contrast brain MRI templates, created from 3T scans of 126 Parkinson's disease patients, with an accompanying set of nine templates of the same MRI contrasts created from 17 healthy controls. The templates are in ICBM152 stereotaxic space, in three different resolutions: 1×1×1 mm^3^, 0.5×0.5×0.5 mm^3^, and 0.3×0.3×0.3 mm^3^ (see Fig. 1, Fig. 2, Fig. 3, Fig. 4).

## 2 Experimental Design, Materials and Methods

### 2.1 Subjects

**Fig. 5:**
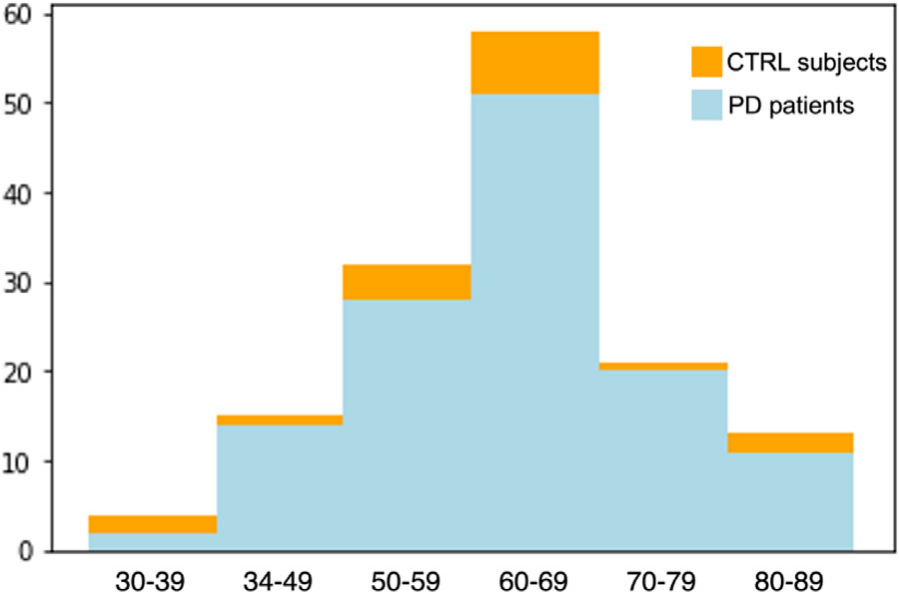
Age distribution of PD patients included in PD126 template (blue), and control subjects included in CTRL17 template (orange).

All Parkinson's patients and healthy controls were recruited through the registry of the Quebec Parkinson Network from 2018 onward and are included in the Montreal Neurological Institute's (MNI) Open Science Clinical Biospecimen Imaging and Genetic (C-BIG) Repository [9]. After rigorous and detailed informed consent, 126 PD patients and 17 controls were scanned, and their age, sex, Unified Parkinson's Disease Rating Scale Part III (UPDRS-III) scores, and Hoen and Yahr (H&Y) scores were recorded. Table 1 shows the demographics of each of the population cohorts. Fig. 5 shows the age distribution for PD patients and control subjects. Note that sex ratios between PD126 and CTRL17 templates differ, resulting in potential male biases in the anatomy of the PD126 template, and female biases in the anatomy of the CTRL17 template. Male anatomical bias in the PD126 template may present as increased cortical and subcortical grey matter atrophy, specifically in the caudate, pallidum and brainstem, compared to female PD patients [10]. Female anatomical bias in the CTRL17 template may present as smaller brain size, and potentially less brain atrophy due to ageing compared to what would be seen in males, specifically relating to peripheral and lateral fissure cerebrospinal fluid volumes and the parieto-occipital region [11].

**Table 1:** Subject demographics for PD126 and CTRL17 templates: sex, age, UPDRS-III scores, and H&Y scores.

Disease status	Sex* (male/female)	Age⁎⁎	UPDRS-III score	H&Y score
PD	82 / 44	64.8 ± 9.1	29.9 ± 15.3⁎⁎⁎	1.8 ± 0.68⁎⁎⁎⁎
controls	4 / 13	62.6 ± 12.7	n/a	n/a

⁎ Sex ratios between PD126 and CTRL17 are different due to recruitment difficulties with control participants during the pandemic.

⁎⁎ Two sample *t*-test revealed no significant differences in age between groups (*p* = 0.97).

⁎⁎⁎ UPDRS-III scores are calculated for 118 PD patients only (those that were available).

⁎⁎⁎⁎ H&Y scores are calculated for 66 patients only (those that were available).

### 2.2 Image acquisition

Each subject was scanned on a Siemens 3T Prisma magnetic resonance imaging (MRI) scanner with the following sequences and accompanying parameters listed in Table 2. From the multi-echo GRE data, several imaging contrasts were created: T2*w, R2* (i.e., 1/T2*), and CLEAR-SWI [3]. All image contrasts were acquired simultaneously at the same imaging session, although it is noted that the image resolutions vary between modalities.

**Table 2:** MRI sequence parameters.

MRI sequence name	Dimension	Relaxation time, TR	Echo time, TE	Flip Angle	Field of view for 2D acquisitions	Slice thickness	3D volumetric resolution	Slice orientation	Matrix size (xyz)	Voxel size
3D T1 Magnetization Prepared - RApid Gradient Echo (MPRAGE)	3D	2300.0 ms	2.98 ms	9 deg	NA	1.0 mm	192 × 256 × 256 mm^3^	Sagittal	192 × 256 × 256	1 × 1 × 1 mm^3^
Proton density (PD) T2-weighted	2D	3000.0 ms	10 ms, 93 ms	165 deg	240 × 240 mm^2^	3.0 mm	NA	T > C-4.0	256 × 256 × 48	0.94 × 0.94 × 3 mm^3^
2D FLuid-Attenuated Inversion Recovery (FLAIR)	2D	9000.0 ms	120.0 ms	165 deg	240 × 240 mm^2^	3.0 mm	NA	T > C-4.0	256 × 256 × 48	0.94 × 0.94 × 3 mm^3^
Multi-echo GRE	3D	30 ms	1.63 ms, 4.10 ms, 6.60 ms, 9.10 ms, 13.00 ms, 16.00 ms, 18.50 ms, 21.00 ms, 23.50 ms, 26.00 ms,	23 deg	NA	0.95 mm	192 × 243 × 243 mm^3^	Sagittal	176 × 256 × 256	0.95 × 0.95 × 0.95 mm^3^
T1-weighted Turbo Spin Echo (neuromelanin)	2D	600.0 ms	10 ms	120 deg	165 × 220 mm^2^	1.8 mm	NA	T > C4.9	240 × 320 × 20	0.7 × 0.7 × 1.8 mm^3^

### 2.3 Image processing and template creation

For each of the imaging modalities, brain masks were generated with BEaST: brain extraction based on nonlocal segmentation technique [12] using the T1w image. After non-local means denoising [5], non-uniformity correction [6], and intensity normalization, each image was registered to stereotaxic space using an in-house PPMI template (which is in ICBM152 stereotaxic space) as the stereotaxic registration target [13]. All imaging modalities for each subject underwent a rigid registration to the T1w image, wherein this transformation was concatenated to the T1w stereotaxic space registration, to bring all registrations into stereotaxic space. Working in a standard space facilitated the creation of T1-T2* fusion images.

T1-T2* fusion MR images were created for each subject following methods described in [2]. Briefly, T1w and T2*w images from the same subject are added together, on a voxel-by-voxel basis, using T1w and T2*w voxel values and spatially varying weights calculated from the R2* volume (i.e., 1/T2*) represented as a brain mask with smoothed susceptibility values to remove values caused by blood vessels. The R2* map weights are inherently larger in the subcortical regions of the brain, and smaller in cortical regions. The voxel-by-voxel fusion calculation is such that higher weights incorporate less of T1w voxel values, and more of T2*w voxel values, resulting in a preservation of T1w cortical contrast, and T2*w subcortical contrast. Improved susceptibility-weighted images (CLEAR-SWI) were created from methods described in [3]. When upsampling FLAIR and PDw modalities, a 3mm blurring kernel was used when calculating between slice interpolation along the z-axis [14], thus these modalities do not have 1mm, 0.5mm or 0.3mm resolution in all axes. All other imaging modalities (T1, T2*w, R2*, T2w, and NM) were ready for template creation.

The T1-T2* fusion images were used to create the transformations that would drive the unbiased average template creation for all imaging modalities, since these images have the best contrast in the cortex from the T1w images and in deep grey nuclei from T2* images, thus the transformations incorporate the most structural information into the deformation for smooth and accurate registrations. Template creation follows the methods from [13], where hierarchical registration using Automatic Nonlinear Image Matching and Anatomical Labelling (ANIMAL) [15] is employed to register an initial template model (here, the MNI PD25 template [2,8] was used) to each of the subjects. This process is performed iteratively, correcting for residual mis-registration at each step, until convergence is reached. Approximately 16 steps were needed for template creation. Once convergence is reached and the unbiased average T1-T2* fusion template is created, the individual subject-to-template transformations are saved and used to create the templates for the remaining contrasts (T1, T2*w, R2*, T2w, PDw, FLAIR, NM, and CLEAR-SWI). Note that all transformations calculated in each hierarchical step of template creation, and from pre-processing steps, are concatenated together to minimize resampling errors. The same transformations were used to create 0.5mm and 0.3mm templates. To create 0.5mm and 0.3mm templates, pre-processed MRI volumes were resampled to 0.3 and 0.5mm resolutions, in stereotaxic space, before being averaged together using the MINC Toolkit [14] to create the templates.

## Ethics Statements

The MR imaging and motor evaluation data collected and shared through the MNI's Open Science C-BIG repository [9] are collected from patients, recruited through the registry of the Quebec Parkinson Network, with a rigorous and detailed informed consent. The de-identified imaging and motor data are collected under protocols that comply with the Health Insurance Portability and Accountability Act of 1996 (HIPAA), the Declaration of Helsinki, and the MUHC Research Ethics Board (C-BIG general protocol: 2017-330, 15-944-MUHC; C-BIG imaging protocol: 2019-4759; QPN protocol: 2015-143, MP-CUSM-NEU-14-053, MP-37-2015-143).

## CRediT authorship contribution statement

**Victoria Madge:** Conceptualization, Methodology, Software, Writing – original draft, Visualization. **Vladimir S Fonov:** Methodology, Software, Data curation, Writing – review & editing. **Yiming Xiao:** Methodology, Software, Writing – review & editing. **Lucy Zou:** Resources. **Courtney Jackson:** Resources. **Ronald B Postuma:** Supervision. **Alain Dagher:** Conceptualization, Supervision, Project administration. **Edward A Fon:** Project administration. **D Louis Collins:** Conceptualization, Methodology, Software, Writing – review & editing, Supervision, Project administration, Funding acquisition.

## Declaration of Competing Interest

The authors declare that they have no known competing financial interests or personal relationships that could have appeared to influence the work reported in this paper.

## Data Availability

Multi-contrast PD126 and CTRL17 templates (Original data) (NITRC). PD126 and CTRL17 (Original data) (MNI).

## Appendix Supplementary materials

Image, application 1

Image, application 2
