# Supplementary material for: A dataset of multi-contrast unbiased average MRI templates of a Parkinson's disease population
Source: Data Brief. 2023 Apr 12;48:109141. doi: 10.1016/j.dib.2023.109141 (PMC10197003; doi:10.1016/j.dib.2023.109141)
Supplement: Supplementary file 2 [file mmc2.pdf]

Montreal Neurological Institute and Hospital

Information and Consent Form

Healthy Volunteer

MONTREAL NEUROLOGICAL INSTITUTE'S OPEN SCIENCE CLINICAL BIOLOGICAL IMAGING AND GENETICS REPOSITORY

Healthy

Title: Montreal Neurological Institute's Open Science Clinical Biological Imaging and Genetics Repository (C-BIG)

Sponsor: Montreal Neurological Institute

C-BIG Directors: Dr. Jason Karamchandani, MD
Dr. Edward Fon, MD, Ph.D.
Dr. Angela Genge, MD
Mr. Alan Evans, Ph.D.

Site: Montreal Neurological Institute and Hospital ("The Neuro")
3801 University Street
Montréal, Quebec H3A 2B4

Preamble

We are inviting you to participate in the Montreal Neurological Institute's Open Science Clinical Biological Imaging and Genetics Repository ("C-BIG"). However, before accepting to participate in this repository and signing the information and consent form, take the time to read, understand and carefully examine the following information.

This form may contain words that you do not understand. We invite you to ask any questions that you may have to a clinical research coordinator ("CRC") associated with the C-BIG, and ask them to explain to you any word or information that is unclear.

What is a " repository"?

A repository is a collection of tissue and fluid samples ("biological material") and personal health information ("data") stored as an organized collection of searchable Samples and/or associated Data stored for specific or as-of-yet unspecified research purposes as a resource for research now and in the future. It provides an important resource for research locally, across Canada, and around the world.

<u>Purpose of the C-BIG</u>

Many diseases affect the brain, spinal cord, peripheral nerves, and skeletal muscle. The ability for physicians to treat patients with neurological disease is often limited by the incomplete understanding of how and why these diseases arise and why they behave the way they do. In order to improve our ability to help treat patients with neurological diseases, we must work to better understand the biology of these diseases and assess how this relates to patient response to the various treatments that can be offered. C-BIG has been created to collect and store biological material and clinical data (including images) to allow us to investigate neurological diseases and human health, and to help us develop new therapies to improve patient care. The Montreal Neurological Institute ("MNI") is an 'Open Science' institute which aims to make material and data available as easily as possible to the research community while protecting the participants who provided it. The institute made this commitment in an effort to accelerate the pace of discovery and the generation of meaningful therapies for neurological disease human disease.

You are being approached as a healthy volunteer, to help us better understand the differences in patients with neurological disease.

<u>Your Contribution to the C-BIG</u>

This section addresses the different contribution that may be sought from a C-BIG participant. Please note that contributions will depend on our state of health.

<u>Clinical data:</u> We are seeking your permission to access your medical health records (medical charts, and electronic medical record including your "Dossier de santé Québec"). The Dossier de Santé Québec is a collection of some of your health care records stored in a provincial database. This clinical information is invaluable, and the combination of this information with your biological materials helps to allow for substantially more meaningful scientific research.

From time to time when relevant for a specific research project performed with C-BIC material/data, CRC may obtain nominal information from institutional clinical repositories such as the MUHC Datawarehouse.

We may also measure your heart rate and blood pressure.

<u>Research data</u>: If you have already participated in research projects, we are seeking your permission to include this data in C-BIG. We are also asking to include in C-BIG the study data from any research projects you will participate in the future. This is only done in agreement with the researcher responsible for the projects where the collection took place.

<u>Questionnaires:</u> We may also ask you to complete questionnaires about your mental and physical health and/or related information such as your quality of life. These questionnaires may ask you to try and solve a puzzle or some other similar sort of cognitive testing. If you agree, we may contact you periodically to request follow-up information.

Imaging data: If you have undergone brain imaging procedures (i.e. MRI, CT-Scan, PET-Scan, MEG) for your clinical care or if you have taken part in brain imaging research, we are seeking your permission to include these images in C-BIG. We are also asking to include in C-BIG the images from any such procedures you will undergo in the future.

Biological material: The precise nature of the biological material you contribute to C-BIG may vary. Listed below are the biological material which will be stored in C-BIG. Many of these materials may not apply to you. For the most part, biological material will only be obtained as part of your normal clinical care and consist of material that is not required for diagnosis and would normally be destroyed.

Your samples may be tested for blood-borne pathogens, which are microorganisms found in the blood and that are capable of causing disease, such as, but not limited to, Epstein-Barr Virus, Hepatitis A, B and C, Herpes viruses, HIV, or Parvovirus B19. If this test is performed and you obtain a positive result, a C-BIG physician will contact you to inform you. If you have a family physician, we may also contact them to arrange for a follow-up and treatment if that is your wish. If you test positive for Hepatitis A, B, C, Human T-Lymphotropic Virus (HTLV), the law requires us to forward your name and identifying information to the Quebec public health authorities. If you test positive for HIV and you are a blood, tissue, or organ donor or recipient, we will do the same. If you test positive for HIV and are NOT a blood, tissue, or organ donor or recipient, we will forward some personal information, but not your name, to the health authorities.

- Blood: You may be asked to provide a blood sample (blood draw). The amount of blood collected will depend on your diagnosis, particularly if you are involved in other research studies. We will ensure that the amount of blood we collect is safe for you. If your care involves surgical intervention, blood will be collected during the surgery if this can be achieved safely. In order to avoid multiple blood draws, if your care or your participation in research projects involves blood draws, we will collect the blood during an otherwise scheduled blood draw.

- Saliva: You may be asked to provide a saliva sample by spitting in a test tube.

- Stool: You may be asked to provide a stool sample.

- Urine: You may be asked to provide a urine sample by urinating into a collection cup.

- Cerebrospinal fluid ("CSF"): Should your clinical care require a lumbar puncture ("LP", or "spinal tap"), or should a LP be part of the procedures in a research study to which you have otherwise consented, a small amount (up to around a tablespoon,15ml, worth of liquid) of additional CSF will be collected for C-BIG. If you are a family member of a participant suffering from a neurological disease (immediate family or other blood relative), you may also be asked to undergo a lumbar puncture for the sole purposes of C-BIG. If this is the case, you will be asked to consent specifically at the time of the procedures.

    - A LP is performed in clinic, by a trained physician. After your back is cleaned, the physician will insert a needle into the space between two vertebrae in your lower back. When the needle is in place, fluid can drip out into a vial. Up to around a tablespoon, 15ml, will be collected. The needle will be removed, the area will be cleaned, and a

bandage will be placed over the needle site. You may be asked to remain lying down for a short time after the procedure.

- Biopsies: If your medical care or your participation in a research project involves a muscle or skin biopsy, a small amount of extra tissue (less than a gram) will be sampled for inclusion in C-BIG. We may also ask adult participants to undergo a muscle or skin biopsy for the sole purpose of C-BIG. If this is the case, you will be asked to consent specifically at the time of the procedures. Biopsies will be performed at the Clinical Research Unit (CRU) at the Neuro by a qualified physician.

    - Muscle biopsy: A small sample of muscle tissue, will be taken for biopsy. Biopsies will be performed at the Clinical Research Unit (CRU) at the Neuro by a qualified physician. This procedure will be done either as a needle biopsy, or as an "open" biopsy. Both procedures use local anesthesia. For needle biopsy procedures, the doctor will insert a needle to your muscle and will take a small sample while following the procedure closely by an ultrasound device. In open biopsies, the physician will make a small incision and take a small amount of muscle tissue.

    - Skin biopsy: You will receive a numbing medicine (anesthetic) on the area where the biopsy will be performed. A skin punch tool will remove a small round piece of skin. If necessary 1 or 2 stiches may be applied (this is not always needed) and a small gauze will be applied to cover the area while it dries. If you have stiches, the doctor may ask you to return to the collection site after 5 to 14 days for removal of the stitches.

- Biomarkers. Biomarkers, including DNA, RNA, Micro-RNA and leucocytes may be extracted from some of your samples stored in C-BIG. DNA is a molecule that contains all the genetic information transmissible from one generation to the next and that provides a series of instructions that determines the characteristics of an individual, such as one's eye color or blood group. Extraction of DNA, RNA and Micro-RNA is optional

- Cell lines. Some of your sample may be used to grow cell lines. Cell lines are cultures of cells that are developed and grown from a single cell obtained from a biological sample. Cell lines are a method to conserve cells that enables the generation of an inexhaustible quantity of these cells for use in subsequent studies. Cell lines can be generated from blood, skin biopsies, muscle biopsies, surgical tissues, and urine, and is optional.

Storage of your Biological Material and Data

Your biological material and data will be identified by a C-BIG code. Only coded biological material and data will be stored in C-BIG. Your identifiable information and the master log (key linking your C-BIG code to your identifiable information) will be kept on a separate, secure, pass-word encrypted server.

Your coded biological material and data will be stored in C-BIG for as long as the C-BIG Governing Board deems them useful for research purposes and can ensure the proper management of repository. C-BIG is housed, in a limited access facility, at the Neuro and is governed by a management

framework (copy available on demand). The C-BIG Governing Board is responsible to ensure that C-BIG is following best practices, is sustainable, and is serving the vision in which it was founded.

We aim to store this biological material and data for as long as they are scientifically valuable. We will be making sure that C-BIG will follow the regulations and standards generally recognized in Canada and in Quebec regarding privacy and that confidentiality is maintained over the years. Should the C-BIG repository be terminated, all the biological material and data will be destroyed in conformity with the policy of the institution.

Future Use of your Biological Material and Data for Research

As previously stated, C-BIG does not perform the research itself. Rather, C-BIG is a resource made available to researchers for the conduct of projects in line with its purpose. Furthermore, C-BIG is one of the tools used to make "Open Science" possible. In this regard, it is essential that the material/data stored in C-BIG be classified in terms of how it will be made accessible to researchers ("level of access"). This is one of the C-BIG Tissue and Data Committee's ("C-BIG TDC") function. When assessing specific material/data's level of access, the C-BIG TDC will take into account your privacy, and consent, and the material/data's level of sensitivity along with the potential risk of its re-identification (how detailed it is, and its potential for misuse). The sharing of C-BIG coded data will be through online databases that are made available to researchers.

Some information will be made publicly available ("Open Access"). This is coded data with very low risk of re-identification and that is not particularly sensitive. This will include data such as summary data about the kinds of diseases included in the collection (ex. 100 participants in C-BIG diagnosed with Parkinson's Disease) as well as the number of participants in C-BIG.

Some information will be made available to researchers who register with C-BIG and make a commitment to follow best-practice ethical standards when examining and analyzing the data ("Registered Access"). This is coded data with a low risk of re-identification and that do not present particular sensitivity. This will include data such as results of basic laboratory analysis, images that were processed with standard tools that avoid re-identification, as well genetic test results that are not sensitive and re-identifiable.

Some information and biological material will only be distributed to researchers whose projects have been approved by the C-BIG TDC and a duly constituted REB. ("Controlled Access"). This is all the data that has not been formally identified by the C-BIG TDC as "Open Access" or "Registered Access" data, and all the biological material, including cell lines from your tissue. Data with a direct or higher risk of re-identification and/or particular sensitivity will always be considered controlled access data. The C-BIG TDC will assess the clinical and scientific validity of the projects wishing to obtained Controlled Access material/data. Before Controlled Access material/data is distributed, a formal agreement will be signed by the researcher and the material/data will be re-coded.

Where possible, the researchers using C-BIG recourses, will be encouraged to return research results from primary experiments to C-BIG following publication.

Potential Harm (Injury, Discomfort or Inconvenience)

Blood Draw

Although drawing blood isn't problematic for the majority of individuals, it could cause bleeding, bruising, discomfort, pain, dizziness or more rarely, infections.

Saliva, Stool and Urine Sample

There are no medical risks in providing a saliva, stool or urine sample.

Skin Punch and Muscle Biopsy

Complications are uncommon following these simple procedures but can occur with any such procedure. Complications associated with skin punch and muscle biopsy include local bleeding and bruising, pain, infection, allergic reaction to the numbing medicine used in the procedure, or damage to the tissues beneath the skin site (such as an artery or a nerve).

Collection of Additional CSF during Lumbar Puncture

There is no known additional medical risk to collecting a few more millilitres (up to around a tablespoon, 15 ml) of CSF during a LP that is performed for you clinical care, or for a research study to which you have otherwise consented.

Possible Financial and Social Risks

When determining the different levels of access to your data, our aim was to reduce the risk of re-identification of the data (that people find out it is about you) and any potential misuse of the data, but we cannot guarantee that this will never occur. As technology advances, there may be new ways of linking data back to you that we cannot foresee today. If your data were re-identified were to fall into the wrong hands, people might misuse information about you and your family's health.

One of the risks associated with genetic research projects is related to the disclosure of your genetic information to outside parties such as employers or insurance companies. Please note that some insurance companies could interpret your participation in this bank (regardless of the results obtained) as a factor that increases your risk even if the study data generated with your DNA is of very limited interest to them. Indeed, the projects conducted with C-BIG biological material, DNA and/or data may include genetic tests performed within the framework of fundamental research. However, if you or a family member has already been diagnosed with a neurological disease and/or has undergone genetic testing for clinical purposes, this information may already be known to the insurance companies that would have asked for this. Measures have been put in place to minimize this risk.

Potential Benefits

You will not directly benefit from your participation in C-BIG. However, the results of the research projects conducted with your biological material and data could benefit individuals suffering from neurological diseases.

Study Results

No individual research results (including genetic information) will be communicated to you unless it becomes the generally recognized applicable standard. This would be, for example, if medical information is found that could be medically useful to you or your family.

Another exception would be if an individual research result housed in C-BIG was one of the inclusion criteria in a research project you are otherwise interested in (see below section on Consent to be re-contacted by the C-BIG Coordinators).

A list of the research projects utilizing C-BIG will be available on the C-BIG's webpage.

Participation / Withdrawal

Your participation in C-BIG is voluntary. You are therefore free to refuse to participate. You may also withdraw from C-BIG at any time, without giving any reason, by informing one of the Directors, the contact person mentioned at the end of this document, or the person listed on the C-BIG's webpage (when it becomes active).

Your decision not to participate in the project or to withdraw from it will have no impact on the quality of care and services to which you are entitled or on your relationship with you treatment team.

If you withdraw from C-BIG, any biological material and data that remain in the bank will be destroyed and a written confirmation will be sent to you. We will be unable to destroy any biological material and data that will have already been distributed for research at the time of your request or information generated from previously distributed material and data.

Protecting Your Health Information

All your biological material and data will be coded and stored in secure rooms and computers. Only double coded biological material and data will be shared for future research. Third parties such as insurers or employers will not have access to C-BIG data unless you provide explicit consent for this access. If your biological material and data is shared internationally, the recipient of your Registered Access and Controlled Access data/material will undertake to provide protection equivalent to the confidentiality rules in effect in Québec and Canada.

In order to ensure the proper management of C-BIG, it is possible that a person or organization mandated by the MUHC REB or the C-BIG Governing Board reviews your C-BIG file and your medical chart as part of an audit. All these individuals and organizations adhere to a policy on confidentiality.

You have the right to access your C-BIG file which includes your identifying information to verify the correctness of the information and have it rectified if it is erroneous.

Compensation/Reimbursement

No monetary compensation is associated with your participation in C-BIG.

It is possible that a commercial product (device or pharmaceutical) may be developed as a result of research conducted with C-BIG resources. You will not receive any compensation if this happens, and you will have no rights nor receive royalties to any products that may be created as a result of these research or any future research studies.

It is possible that the patient community at large benefits as a consequence of your contribution. Your donation will hopefully result in new scientific insight that will be made available to scientific community with the aims of innovating and improving patient care. A list of the publications generated as a consequence of C-BIG will appear on the C-BIG website.

Consent to be re-contacted by the C-BIG coordinators

It is possible that on the basis of research studies performed on your biological material and data that a principal investigator / clinician scientist would like to re-contact you for research purposes. You can elect to be available for re-contact should you so choose.

In case of Injury

If you should suffer any injury following any procedure related to C-BIG, you will receive the appropriate care and services for your medical condition.

By accepting to participate in C-BIG, you are not waiving any of your legal rights nor discharging the administrators, the sponsor and the institution, of their civil and professional responsibility.

Ethical considerations

The C-BIG repository has been approved by the MUHC REB. The MUHC REB is responsible for its ethics monitoring.

Funding of C-BIG

The C-BIG repository is funded by the MNI. The MNI will operate on a cost recovery basis. As determine by the C-BIG TDC on a project by project basis, fees may be collected from a subset of internal funded projects, and outside researchers and industry collaborators employing C-BIG biological material and data in their research. Your biological materials will not be sold nor made exclusively available to a commercial entity where the project does not meet the C-BIGs goal of advancing our understanding of human disease and improving patient care.

Contact Information

If you have questions concerning C-BIG, or if you have a problem you think may be related to your participation in the C-BIG, you may communicate with the associate director of the C-BIG repository. This information is available on the C-BIG website (https://mcgill.ca/c-bigneuro/).

For any questions concerning your rights as a participant in the C-BIG or if you have comments or wish to file a complaint, you may communicate with the MUHC Patient Ombudsman at the following phone number: **514 934 1934, 22223**

(To be filled out by the clinical research coordinator)

I am being asked to provide:

	Yes	No
Blood	☐	☐
Skeletal Muscle	☐	☐
Cerebrospinal Fluid	☐	☐
Skin Biopsy	☐	☐
Saliva Sample	☐	☐
Urine Sample	☐	☐
Stool Sample	☐	☐
MRI, MEG, PET images for the sole purpose of C-BIG	☐	☐

STATEMENT OF CONSENT

Montreal Neurological Institute's Open Science Clinical Biological Imaging and Genetics Repository

I- Signature of the C-BIG Participant

I have reviewed this information and consent form. The C-BIG and the terms of this information and consent form were explained to me. My questions were answered and I was given sufficient time to make a decision. After reflection, I consent to participate in C-BIG in accordance with the conditions stated above.

	Please check box Yes	No
I agree to be re-contacted by e-mail for follow-up information on C-BIG (otherwise information will be made available on the C-BIG website)<br>If so, my e-mail address is: ____________________	☐	☐
I agree for the clinical research co-ordinators to access my *dossier de Santé du Quebec* or institutional clinical repository to obtain clinical, laboratory, and radiological data relevant for C-BIG periodically and/or as needed.	☐	☐
I agree that my samples be used to extract DNA, RNA and micro-RNA	☐	☐
I agree that my sample be used to generate cell lines	☐	☐
I agree to be re-contacted to complete additional questionnaires about my health and quality of life.	☐	☐
I agree to be re-contacted for additional sampling (blood, skin and muscle biopsy).	☐	☐
I agree to be re-contacted for future specific research projects if deemed warranted by the project's principal investigator / clinician.	☐	☐
I agree to have my study data from any other research project stored in C-BIG	☐	☐

____________________	____________________	____________________
Participant's name	Participant's signature	Date

II. Signature of the person who obtained consent

I have explained the terms of the present information and consent form to the research participant and I answered all his/her questions.

Name of the person who obtained consent	Signature of the person who obtained consent	Date
